# The enhancement effect of mungbean on the physical, functional, and sensory characteristics of soy yoghurt

**DOI:** 10.1038/s41598-024-54106-9

**Published:** 2024-02-14

**Authors:** Gyeongseon An, Sunghoon Park, Jungmin Ha

**Affiliations:** 1 Department of Plant Science, Gangneung-Wonju National University, Gangneung, 25457 Republic of Korea; 2 Haeram Institute of Bakery Science, Gangneung-Wonju National University, Gangneung, 25457 Republic of Korea; 3 Department of Food & Nutrition, Gangneung-Wonju National University, Gangneung, 25457 Republic of Korea

**Keywords:** Plant sciences, Nutrition

## Abstract

Vegetable drinks offer a convenient way to increase the daily intake of vegetables containing vitamins, antioxidants, and fiber. In this study, we discovered that mungbean milk serves as a carbohydrate source during fermentation using lactic acid bacteria (LAB) and enhances the nutritional value of vegetable yoghurt. Mungbean milk reduces pH while titratable acidity increases faster than soybean milk during fermentation. M0S, Soybean milk 100% with added sucrose exhibited the highest titratable acidity after 16 h of fermentation. The acetic acid content of all samples did not show significant changes during fermentation, but the lactic acid content increased. Proximate analysis showed no significant change during fermentation, regardless of the fermentation time and mixing ratio of mungbean to soybean milk. The sucrose content of samples except M0S decreased after 16 h of fermentation. Mungbean milk exhibited high antioxidant activity both before and after fermentation, while M0S showed the lowest antioxidant activity. The results of this study demonstrated the potential application of mungbean milk to improve fermented vegetable drinks using LAB functionally. Fermented mungbean milk yoghurt can be a valuable addition to a healthy and balanced diet for those who consume plant-based diets.

## Introduction

The consumption of animal food is gradually increasing because of the recent growth in human population^1^. This trend has been observed in developed as well as developing countries^2^. Although animal food is an important nutrition source for humans, especially because of its high protein content, there have been many problems, such as bioethics, greenhouse gas emissions in livestock, the slaughtering process, and the lactose intolerance in dairy products. Plant-based foods can be a great alternative to animal foods, and currently, the demand for plant-based foods is gradually increasing^3,4^.

Soybean milk is a substitute for consumers who cannot drink cow’s milk because of a milk protein allergy or lactose intolerance. Soybean (*Glycine max* (L.) Merrill) is a legume crop with a high protein content (approximately 40%) that can serve as an inexpensive protein source^5–7^. Soybean milk has a similar protein content to cow’s milk but provides fewer calories^8^. β-conglycinin and glycinin account for more than 70% of soybean protein^9^. Although soybean milk may cause indigestion due to oligosaccharides such as raffinose and stachyose, the use of lactic acid bacteria (LAB) for breakdown of carbohydrate can address this issue. In addition to soybeans, various legumes, such as peanuts and red beans, can be used as food ingredients. LAB fermentation of these legumes can improve food quality by reducing the beany flavor and increasing antioxidant activity^10,11^.

Mungbeans (*Vigna radiata* (L.) R. Wilczek) are rich in vitamins and minerals. In addition, mungbeans have a higher carbohydrate (approximately 62.3%) and lower fat (approximately 1.9%) content than soybeans, which contain ~ 33.9% carbohydrates and ~ 21% fat^12–14^. Total phenolic content and antioxidant activity in mungbean is comparatively higher than in soybean. Moreover, mungbean has higher tyrosinase inhibition than other legume crops, which can prevent type II diabetes^15,16^. However, despite these advantages, only fewer studies on fermented foods using mungbeans have been conducted. In particular, changes in physiochemical properties, such as organic acid and sugar contents, during fermentation have not been studied in fermented mungbean milk.

We hypothesized that mungbean milk, which has a higher carbohydrate content than soybean milk, can be an efficient substrate for LAB. To test this hypothesis, vegetable yoghurts with different ratios and fermentation times of mungbean and soybean milk were prepared. The fermentation characteristics were evaluated using physicochemical analysis and sensory tests. The results of this study contribute valuable information on the applicability of fermented mungbean milk as a functional food.

## Materials and methods

### Chemicals and reagents

NaOH and H_2_SO_4_ were acquired from DAEJUNG (Korea). Ultrapure water and acetonitrile were obtained from Thermo Fisher Scientific (USA). Acetic acid, lactic acid, and 2,2’-azino-bis-3-ethylbenzothiazoline-6-sulfonic acid (ABTS) were purchased from Sigma-Aldrich (USA), and sugar standards (glucose, fructose, sucrose) were sourced from Chemfaces (China). Potassium persulfate was bought from YAKURI (Korea), and phosphate-buffered saline was obtained from GeneAll (Korea). In the case of 2,2-diphenyl-1-picrylhydrazyl (DPPH), it was procured from BIOMAX (Korea).

### Sample preparation

Soybean (*Glycine max* cv. Daewonkong) and mungbean (*Vigna radiata* cv. VC1973A) (provided by Crop Genomics Lab in Seoul National University, Seoul, South Korea) were harvested at the Gangneung-Wonju National University Experimental Farm in Gangneung, South Korea (37.77°N, 128.86°E). The plant collection and use was in accordance with all the relevant guidelines. For fermentation, a commercial kefir yoghurt starter (Lallemand, France) containing *Lactococcus lactis*, *Lactococcus cremoris*, *Lactococcus diacetylactis, Lactobacillus acidophilus*, *Saccharomyces cerevisiae*, *Kluyveromyces lactis*, kefir grains, maltodextrin, and milk was used. The fermentation was carried out at 25 °C according to the manufacturer's protocol.

### Vegetable yoghurt preparation and fermentation

After rinsing with distilled water, 100 g of mungbean and soybean seeds were soaked overnight in 2 L of distilled water at room temperature. The soaked seeds were ground with 1 L of distilled water at 70 °C. The resulting slurry was filtered using cheesecloth, and thereafter the supernatant was heated at 95 ± 2 °C for 20 min to obtain mungbean and soybean milk.

Six samples (M0, M25, M50, M75, M100, and M0S) were prepared by varying the mixing ratios of mungbean and soybean milk. To validate the role of sucrose in fermentation, 3.5 g of sucrose was added to 100% soybean milk (M0S) (Table 1). Per 100 mL of each sample, 0.3 g of yoghurt starter was added, and the samples were fermented at 25 °C for 0, 8, and 16 h.

**Table 1:** Mixing ratio of mungbean to soybean milk.

Samples*	M0	M25	M50	M75	M100	M0S
Soybean milk (mL)	100	75	50	25	0	100
Mungbean milk (mL)	0	25	50	75	100	0
Sucrose (g)	0	0	0	0	0	3.5

*M0, 25, 50, 75, and 100 indicate the content (%) of mungbean milk, and M0S indicates soybean milk 100% with sucrose.

The yoghurt samples were freeze-dried for 72 h. Freeze-dried samples were extracted with 70% ethanol for 24 h and filtered with a 0.4 μm syringe filter for analysis.

### pH and titratable acidity (TA)

The pH of each sample was measured using a pH meter (OHAUS ST2100-F; Parsippany, USA). Titratable acidity (TA) was measured according to the method described by Jung et al.^17^. The sample and distilled water were mixed in a ratio of 1:1 and titrated by adding 0.1 N NaOH until the pH reached 8.3. At this time, the TA (%) was calculated according to the following. formula:

$$Acidity \,\left(\%\right)=\frac{0.1\ N \,NaOH\left(ml\right) \times factor \times dilution \,rate \times 0.009}{Sample(ml)}\times 100$$

### Sugar and organic acid analysis

Sugars and organic acids in vegetable yoghurt were measured using an ultra-performance liquid chromatography system (Shimadzu, Japan) equipped with MPM-40, SCL-40, DGU-405, LC-40D xs, RID-20A, SPD-M40, CTO-40C, and SIL-40C xs. The sample was filtered using a Sep-Pak C18 cartridge (WAT043995, Waters, Ireland) before sugar analysis. For monosaccharide and sucrose content analysis, a ZORBAX carbohydrate column (5 μm, 4.6 mm × 150 mm, Agilent, USA) was used. The monosaccharide and sucrose contents were quantified using a refractive index detector. The mobile phase consisted of 25% ultrapure water (solvent A) and 75% acetonitrile (solvent B) at a flow rate of 1.5 mL/min for 10 min.

Acetic acid and lactic acid were separated using ZORBAX SB-C18 columns (3.5 μm, 4.6 mm × 150 mm, Agilent, USA). For the mobile phase, the methods of Wang et al. and Granata et al. were used with minor modifications^18,19^. To detect acetic and lactic acid, we initially used a mobile phase consisting of 85% 0.01 N H_2_SO_4_ (solvent A) and 15% acetonitrile (solvent B). The maximum mobile phase consisted of 50% 0.01 N H_2_SO_4_ and 50% acetonitrile. The mobile phase was passed through the column for 30 min at a flow rate of 0.5 mL/min. The photodiode array detector was set to detect the presence of acetic and lactic acids at a wavelength of 210 nm. For sugar and organic acid analysis, the injection amount of the sample was 50 μL and 5 μL, respectively. For both analyses, the column oven temperature was set at 40 °C, and the measurement was repeated three times. Standard curves were constructed using glucose, fructose, sucrose, acetic acid and lactic acid as standards.

### Physicochemical analysis

#### Viscosity measurement

The viscosity of the yoghurt was measured using a rheometer (MCR 302, Anton Paar, Austria) with a plate-cone geometry (CP50-1). The shear rate was increased from 0.01 to 1000 s^−1^, and the viscosities at a shear rate of 10 s^−1^ were measured. All measurements were repeated three times at 25 °C.

#### Proximate analysis

The crude protein, crude fat, and ash contents of mungbean and soybean milk yoghurt according to fermentation time were measured at the East Coast Marine Biological Resources Research Center. Crude protein, crude fat, and ash content were measured using methods in accordance with the AOAC^20^. All measurements were repeated three times.

#### Sensory test

Commercially available vegan Greek yoghurt products (CP, Yogutdoo, Korea, https://smartstore.naver.com/yogutdoo) and M0, M50, M100, and M0S samples fermented for 0 and 8 h were used for sensory evaluation. The sensory test was conducted on 37 students from the Department of Food & Nutrition at Gangneung-Wonju National University and evaluated using a 5-point scale with a minimum of 1 point and a maximum of 5 points for color, flavor, sweetness, texture, sourness, beany flavor, and overall preference.

### Antioxidant capacity

#### ABTS radical scavenging activity

The radical scavenging activity of 2,2’-azino-bis-3-ethylbenzothiazoline-6-sulfonic acid (ABTS) was measured using the method of Jo et al*.*^21^ with modifications. Ascorbic acid (012–04,802, FUJIFLIM, Japan) was used as the standard (0, 10, 20, 40, 60, 80, and 100 mg/L). The ABTS solution (10102946001, Sigma-Aldrich, USA) and potassium persulfate (28718, YAKURI, Korea) were mixed at a ratio of 1:1 (v/v) and stored at room temperature for 24 h. The solution was then diluted in phosphate-buffered saline (SM-P04-100, GeneAll, Korea), and the absorbance at 740 nm was adjusted to 0.7 ± 0.03. For activity measurement, 20 µL of each sample was added to a 96-well plate, and 20 μL of ascorbic acid and 180 μL of ABTS mixture solution were added. After 10 min in the dark, absorbance was measured at 740 nm using a microplate reader (51119000, Thermo Fisher Scientific, USA).

#### DPPH radical scavenging activity

The OxiTec™ DPPH Antioxidant Assay Kit (BIOMAX, Korea) was used to measure 2,2-diphenyl-1-picrylhydrazyl (DPPH) radical scavenging activity, following the manufacturer’s protocol. Twenty μL of Trolox (0, 10, 20, 40, 60, and 80 mg/L) was used as standard. To 20 μL of each sample in triplicate, 80 μL of assay buffer and 100 μL of DPPH working solution were added. After 30 min in the dark, the absorbance was measured at 520 nm using a microplate reader (51119000, Thermo Fisher Scientific, USA).

### Statistical analysis

All preparation experiments were repeated at least three times, and their properties were analyzed. Results are presented as the mean values ± standard deviation (SD) of at least three repeats. All parameters were validated for normality (Shapiro–Wilk test) and homogeneity of variance (Levene's test). Statistical significance was determined using analysis of variance and Duncan's multiple range test, and differences were considered significant at *p* < 0.05.

## Results and discussion

### Changes in pH and TA during fermentation

**Figure 1:**
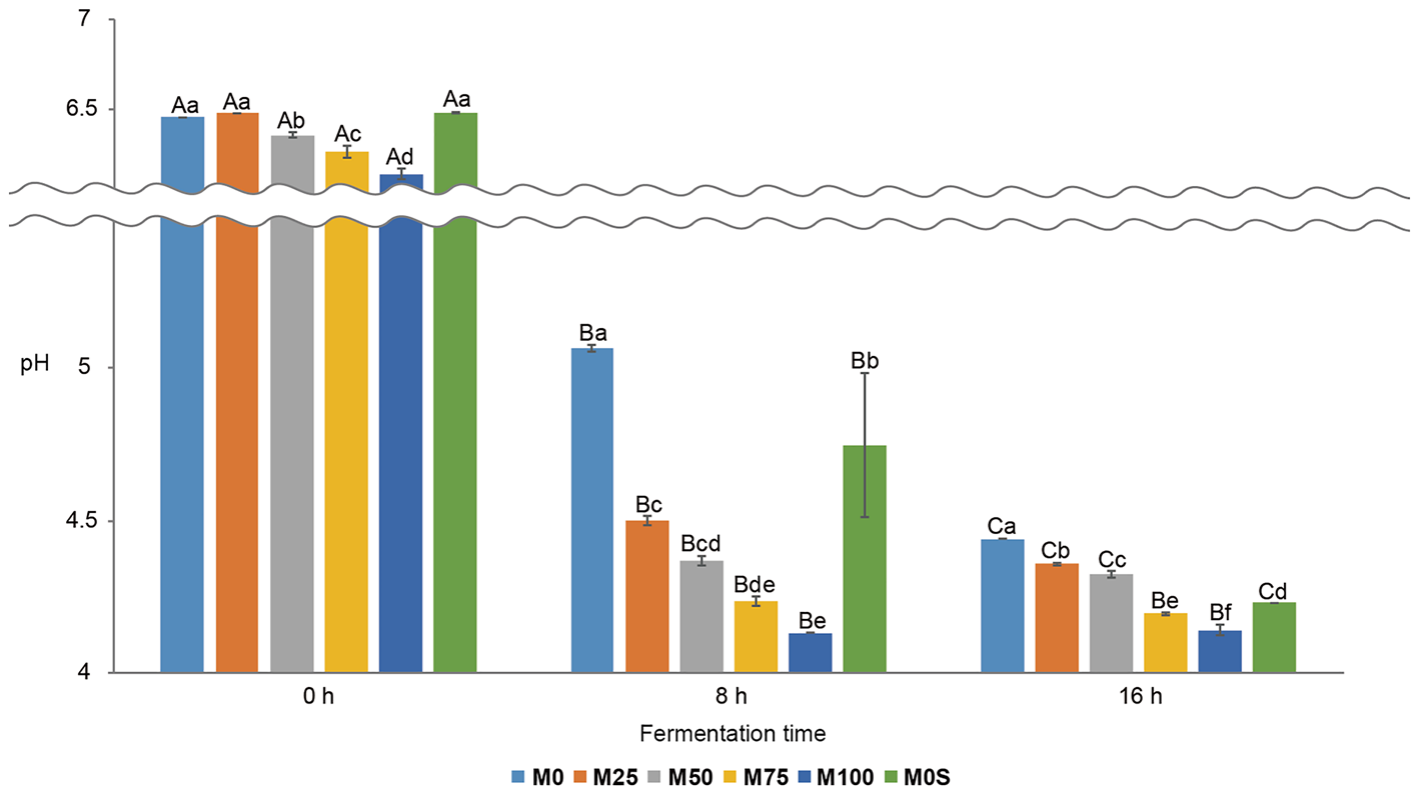
Changes in pH of vegetable yoghurts with different ratios of mungbean and soybean milk during fermentation. 0 h, 8 h, and 16 h represent the fermentation times. M0, 25, 50, 75, and 100 represent the mungbean milk content (%), and M0S represents soybean milk 100% (mungbean milk 0%) with sucrose added. Letters indicate statistically significant differences in pH value using Duncan’s multiple test (*p* < 0.05). Error bars indicate the standard deviation. Uppercase letters indicate significant differences in pH depending on the fermentation time in each sample, and lowercase letters indicate significant differences in pH depending on the mixing ratio in each fermentation time.

Depending on the mixing ratio of mungbean to soybean milk (M0, M25, M50, M75, M100, and M0S), changes in pH showed significant differences according to fermentation time (0, 8, and 16 h) (Fig. 1). M0 had the highest pH among fermentation samples with pH 5.07 ± 0.01 after 8 h of fermentation, and pH 4.44 ± 0.00 after 16 h of fermentation. M100 had pH 6.14 ± 0.03 before fermentation (0 h), pH 4.13 ± 0.00 after 8 h of fermentation, and pH 4.14 ± 0.02 after 16 h of fermentation, the lowest among all fermentation times. As the proportion of mungbean milk increased (M25, M50, M75 and M100), the pH significantly decreased after 8 h of fermentation compared to soybean milk (M0 and M0S), indicating that adding mungbean milk may promote fermentation by LAB. The high carbohydrate content of mungbean milk may result in a decreased pH after fermentation. Sucrose was introduced to soybean milk 100% to examine the role of carbohydrates in LAB fermentation. There was no significant difference in pH between M0 (6.46 ± 0.00) and M0S (6.48 ± 0.00) before fermentation, whereas M0S showed significantly lower pH than M0 after fermentation (Fig. 1), indicating that adding sucrose can promote fermentation by LAB. Therefore, introducing carbohydrates can enhance fermentation by LAB.

**Figure 2:**
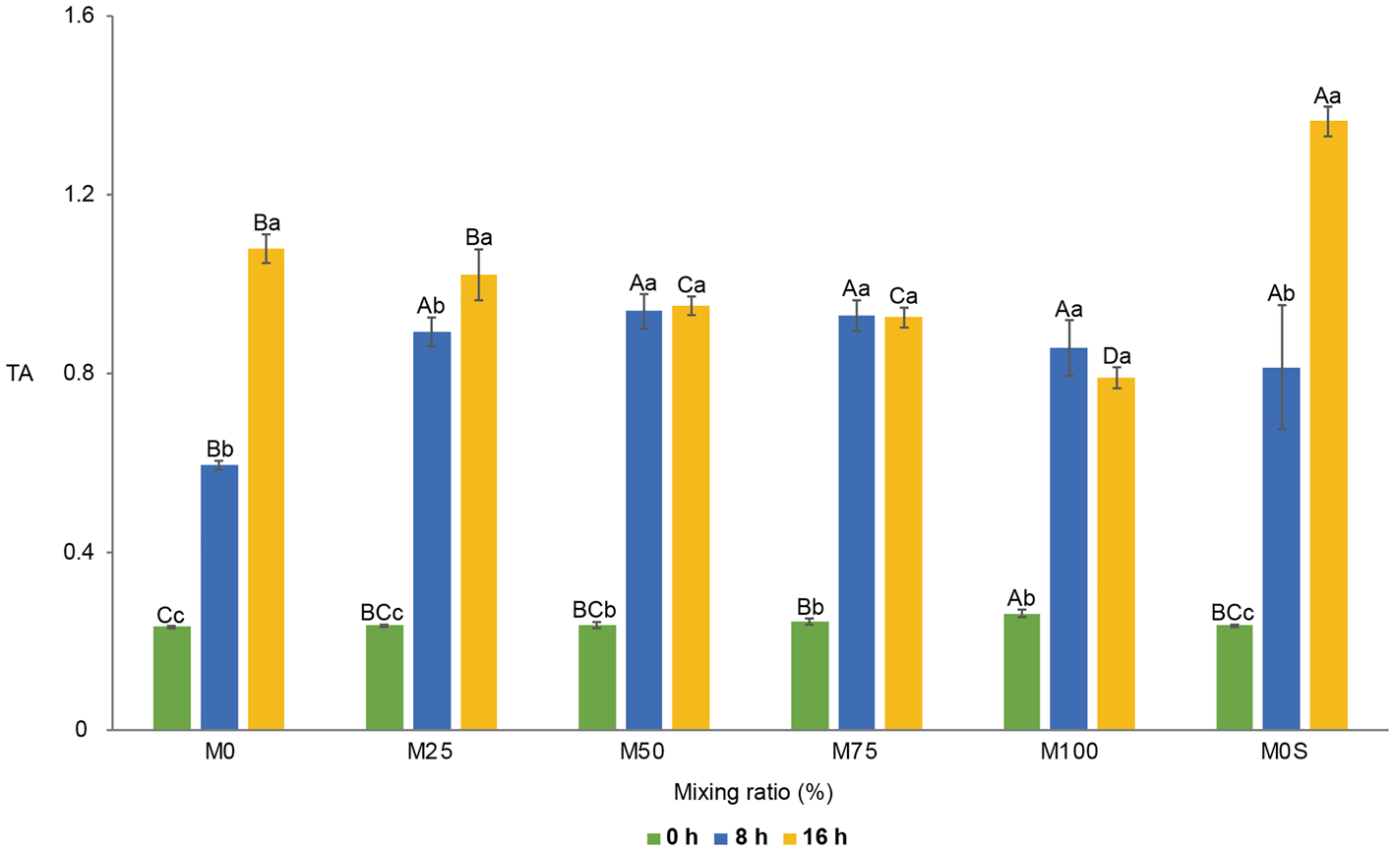
Changes in TA of vegetable yoghurts with different ratios of mungbean and soybean milk during fermentation. M0, 25, 50, 75, and 100 represent the mungbean milk content (%), and M0S means soybean milk 100% (mungbean milk 0%) with sucrose added. Uppercase and lowercase letters indicate statistically significant differences in TA value depending on the mixing ratio and fermentation time, respectively, using Duncan’s multiple test (*p* < 0.05). Error bars indicate the standard deviation. A–D represent significant differences in the TA of the samples according to each fermentation time, and a–c represent significant changes in TA as the fermentation time of each sample increases.

TA ranged from 0.23% ± 0.00% to 0.26% ± 0.01% before fermentation but significantly increased to 0.60% ± 0.01% to 0.94% ± 0.04% after 8 h of fermentation for all samples (Fig. 2). In M0, TA increased gradually over 16 h but increased rapidly when the proportion of mungbean milk increased. Therefore, at 8 h, M0 showed a significant but minimal change in TA among the samples. Samples with a mungbean milk content of 50% or more showed no significant change in TA between 8 and 16 h of fermentation. For M0S, TA increased as fermentation time increased, showing the highest TA (1.37% ± 0.03%) at 16 h (Fig. 2).

These findings were consistent with the results of previous studies on the fermentation of vegetable beverages^22–24^. During fermentation, LAB produce organic acids such as lactic and acetic acid by decomposing carbohydrates for energy, decreasing pH and increasing TA^18^. The study results show that fermentation by LAB can be efficiently promoted when mungbean milk or sucrose is added, providing carbohydrates to LAB to produce vegetable yoghurt.

### Physical characteristics of vegetable yoghurt

**Figure 3:**
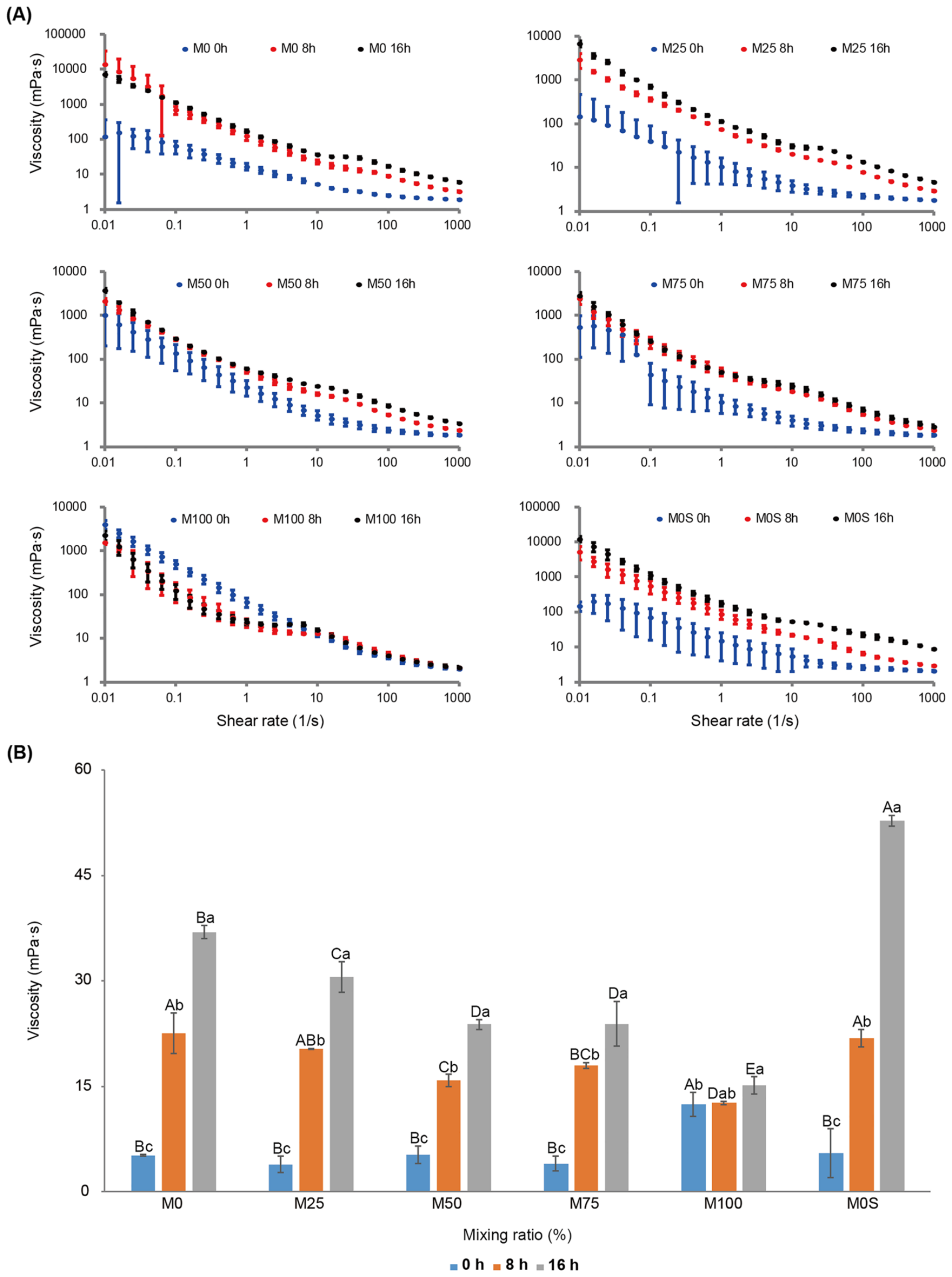
Viscosity of vegetable yoghurt depending on the ratio of mungbean to soybean milk. (**A**) The change in viscosity of vegetable yoghurt according to the increase in shear rate was measured. Blue, red, and black dots indicate 0 h, 8 h, and 16 h of fermentation, respectively. (**B**) The change in viscosity of all samples according to the fermentation time (0 h, 8 h, and 16 h) based on the shear rate of 10 s^-1^. M0, 25, 50, 75, and 100 represent the mungbean milk content (%), and M0S means soybean milk 100% (mungbean milk 0%) with sucrose added. Letters indicate statistically significant differences in viscosity value using Duncan’s multiple test (*p* < 0.05). Error bars represent standard deviation. A–E represent significant differences in viscosity of the samples based on fermentation time, and a–c represent significant changes in viscosity as the fermentation time of each sample increases.

Changes in the viscosity of vegetable yoghurt were measured according to shear rate (Fig. 3A). All yoghurt samples showed shear-thinning behavior, indicating that their viscosity decreased with the shear rate^25^. As the fermentation time increased, the viscosity of samples increased from 12.63 ± 0.25 to 22.57 ± 2.90 mPa·s after 8 h of fermentation and from 15.13 ± 1.29 to 52.83 ± 0.76 mPa·s after 16 h of fermentation (Fig. 3B). In general, during fermentation, the viscosity increased in all samples, as previously reported for soybean milk^26^. The effect of fermentation on viscosity decreased significantly with an increase in the proportion of mungbean milk. After 16 h of fermentation, M0 showed a significantly higher increase in viscosity than other samples containing mungbean milk, consistent with a previous study^27^. M0S had the highest viscosity value of 52.83 ± 0.76 mPa·s after 16 h of fermentation. Considering sucrose is one of the most preferred Carbohydrates for LAB, it could be broken down into lactic acid during fermentation^28,29^. Conversely, M100 showed the least change in viscosity after fermentation.

Proteolysis during fermentation can result in the coagulation of vegetable beverages. Therefore, changes in viscosity during fermentation may be caused by LAB proteolytic activity. The proteolytic ability and specificity vary depending on the type of LAB. Aguirre et al.^30^ reported that the LAB strain had vigorous proteolytic activity against soybean proteins, especially β-conglycinin, which is the major component of soybean protein. Mungbean protein is mainly composed of globulin and albumin^31,32^. M0 showed a significantly higher increase in viscosity during fermentation than M100 (Fig. 3B). For proximate analysis in this study, M100 had the lowest crude protein of 1.70 ± 0.00 g, and M0 had the highest of 2.90 g ± 0.00 g (Supplementary Table 1). Therefore, the reason for the decrease in viscosity when the portion of mungbean milk increased is likely because mungbean contains less protein than soybean^33,34^.

Overall, the increase in the viscosity of vegetable yoghurt is likely caused by fermentation, resulting in proteolysis by LAB, and the ratio of soybean to mungbean milk affects the viscosity range after fermentation.

### Changes in organic acid and sugar contents of vegetable yoghurt

The acetic acid and lactic acid contents were measured by varying the mungbean and soybean milk ratio and the fermentation time (Table 2). The lactic acid content ranged from 4.32 ± 0.82 ppm to 17.54 ± 2.67 ppm before fermentation (0 h) and 144.68 ± 32.47 to 225.63 ± 1.60 ppm after 16 h of fermentation. For all samples, the lactic acid content increased significantly as fermentation time increased. The acetic acid content showed no significant differences during fermentation in any of the samples. Although acetic and lactic acids are major by-products of fermentation by LAB, their production and quantities can vary depending on the strain of LAB^35–38^. In this study, lactic acid was the major product of the fermentation process of mungbean and soybean milk by LAB, resulting in pH and TA changes (Figs. 1 and 2).

**Table 2:** Comparison of organic acid (acetic and lactic acid) contents of vegetable yoghurt according to the ratio of mungbean to soybean milk and fermentation time.

Samples		Acetic acid (ppm)	Lactic acid (ppm)
M0	0 h	3.45 ± 0.93^N.S^	17.54 ± 2.67^c^
	8 h	2.00 ± 0.25^N.S^	61.79 ± 0.88^b^
	16 h	2.52 ± 0.52^N.S^	209.05 ± 1.39^a^
M25	0 h	2.88 ± 0.57^N.S^	16.03 ± 1.19^c^
	8 h	1.91 ± 0.54^N.S^	68.41 ± 3.71^b^
	16 h	1.44 ± 0.64^N.S^	225.63 ± 1.60^a^
M50	0 h	1.99 ± 0.67^N.S^	14.17 ± 1.36^c^
	8 h	2.89 ± 0.80^N.S^	66.93 ± 1.45^b^
	16 h	1.61 ± 1.07^N.S^	186.79 ± 1.36^a^
M75	0 h	2.27 ± 0.90^N.S^	9.39 ± 0.84^c^
	8 h	2.34 ± 0.46^N.S^	68.74 ± 1.15^b^
	16 h	2.14 ± 1.02^N.S^	198.36 ± 1.38^a^
M100	0 h	1.62 ± 0.68^N.S^	4.32 ± 0.82^c^
	8 h	1.65 ± 1.16^N.S^	66.68 ± 1.45^b^
	16 h	2.11 ± 0.93^N.S^	191.78 ± 1.29^a^
M0S	0 h	2.28 ± 0.54^N.S^	6.59 ± 0.56^c^
	8 h	2.39 ± 0.24^N.S^	45.99 ± 1.87^b^
	16 h	1.93 ± 0.97^N.S^	144.68 ± 32.47^a^

*N.S: not significant.

M0, 25, 50, 75, and 100 represent the mungbean milk content (%), and M0S represents soybean milk 100% (mungbean milk 0%) with sucrose added. Fermentation times are represented by 0 h, 8 h, and 16 h. Letters indicate statistically significant differences between the acetic and lactic acid contents, as determined using Duncan’s multiple test (*p* < 0.05). a–c illustrate significant changes in acetic and lactic acid contents as the fermentation time of each sample increased. The units of acetic and lactic acid contents are ppm.

Glucose, fructose, and sucrose in soybean milk are energy sources that can be used for LAB growth^39^. Sucrose is a disaccharide consisting of glucose and fructose monosaccharides. Changes in monosaccharide and sucrose contents were measured according to fermentation time (Table 3). Generally, the sucrose content of vegetable yoghurt decreases during fermentation. For soybean milk, after 16 h of fermentation, M0 showed a decrease in sucrose content compared to before fermentation. However, for M0S, the sucrose content after 16 h of fermentation was higher than before fermentation, possibly due to differences in solubility resulting from unintentional temperature variations before and after fermentation. The yoghurt samples were prepared at room temperature (18–20 °C), while fermentation was conducted at 25 °C. Although the temperature difference before and after fermentation initially led to a temporary increase in sucrose, M0S exhibited a lower sucrose content after 16 h of fermentation compared to 8 h. When mungbean milk was added, the sucrose content significantly decreased during fermentation. This indicates that the sucrose content of soybean milk and mungbean milk decreased because of the degradation of disaccharides by LAB during fermentation, and that mungbean milk can be a substrate for LAB.

**Table 3:** Analysis of changes in monosaccharides (glucose, fructose) and sucrose in vegetable yoghurt before and after fermentation according to the ratio of soybean to mungbean milk.

Samples		Glucose (ppm)	Fructose (ppm)	Sucrose (ppm)
M0	0 h	113.33 ± 9.33^b^	162.29 ± 12.16^b^	1710.80 ± 36.25^a^
	8 h	57.51 ± 18.53^c^	191.70 ± 25.60^b^	1859.00 ± 113.33^a^
	16 h	268.06 ± 24.04^a^	287.73 ± 32.93^a^	1117.68 ± 35.16^b^
M25	0 h	108.41 ± 3.17^N.S^	314.32 ± 17.67^a^	1533.45 ± 137.43^a^
	8 h	112.09 ± 6.08^N.S^	242.01 ± 27.41^b^	1447.42 ± 172.25^a^
	16 h	136.60 ± 26.63^N.S^	216.71 ± 28.75^b^	1136.68 ± 107.52^b^
M50	0 h	245.39 ± 15.38^a^	283.03 ± 68.73^a^	1719.22 ± 48.58^a^
	8 h	141.64 ± 13.40^c^	153.63 ± 56.20^b^	1089.69 ± 119.31^b^
	16 h	209.92 ± 13.56^b^	277.37 ± 6.53^a^	804.97 ± 156.03^c^
M75	0 h	373.12 ± 16.56^a^	263.49 ± 33.66^a^	1418.35 ± 203.46^a^
	8 h	163.30 ± 0.95^b^	125.26 ± 26.07^b^	702.15 ± 5.04^b^
	16 h	183.92 ± 12.96^b^	224.32 ± 53.60^a^	311.82 ± 9.75^c^
M100	0 h	332.16 ± 27.10^a^	222.90 ± 34.44^a^	397.34 ± 3.58^a^
	8 h	225.00 ± 3.34^b^	96.57 ± 2.11^b^	194.83 ± 14.44^c^
	16 h	256.93 ± 40.70^b^	179.23 ± 16.93^a^	255.50 ± 18.99^b^
M0S	0 h	66.34 ± 9.75^b^	110.11 ± 16.05^b^	10,963.77 ± 57.09^c^
	8 h	40.56 ± 3.23^b^	134.66 ± 35.32^b^	13,669.69 ± 113.38^a^
	16 h	541.07 ± 91.18^a^	273.27 ± 48.35^a^	12,571.20 ± 114.06^b^

*N.S: not significant.

M0, 25, 50, 75, and 100 represent the mungbean milk content (%), and M0S means soybean milk 100% (mungbean milk 0%) with sucrose added. Fermentation times are represented by 0 h, 8 h, and 16 h. Letters indicate statistically significant differences in monosaccharides (glucose and fructose) and sucrose values using Duncan’s multiple test (*p* < 0.05). a–c show the significant sugar change that occurred as the fermentation time of each sample increased. The unit of sugar content is ppm.

The monosaccharide content of mungbean and soybean milk did not show the same trend during fermentation. The fructose content in M0 and M0S increased after 16 h of fermentation. When the proportion of mungbean milk was higher than 50% (M50, M75, and M100), the fructose content decreased for the first 8 h and increased significantly after 16 h. The glucose content in M0 and M0S increased significantly after 16 h of fermentation, whereas it decreased in general during fermentation when mungbean milk was added.

During fermentation, microorganisms hydrolyze sucrose for their growth^40^. This process increases the contents of monosaccharides, such as glucose and fructose, in soybean milk (M0 and M0S), consistent with previous studies that the hydrolysis of α- and β-galactosidases is catalyzed and increases the monosaccharide content during fermentation^35,41^. Elghali et al.^35^ reported that cultivating *Lactobacillus plantarum* and *L. casei*, in soybean milk increased the glucose and fructose contents significantly after fermentation. However, in this study, the monosaccharide content changed differently depending on the ratio of mungbean to soybean milk. Santos et al.^41^ reported that glucose content increased up to 8 h of fermentation when several combinations of mixed strains were cultured in peanut and soybean milk. Among them, the glucose content of the mixed strain of *Pediococcus acidilactici* and *Saccharomyces cerevisiae* decreased at 16 h of fermentation, but increased at 24 h of fermentation. The monosaccharide content can increase because of the hydrolysis of polysaccharides and disaccharides during fermentation^42^. Depending on the rates of consumption of monosaccharides and the hydrolysis of polysaccharides and disaccharides into monosaccharides, the monosaccharide content may increase or decrease. The yoghurt starter used in this study contained several strains, such as *L. acidophilus* and *Lactococcus lactis*, each of which may have a different growth rate during fermentation, resulting in different sugar consumption or hydrolysis rates during fermentation. Therefore, in this study, fructose and glucose contents were less likely to have specific trends depending on the fermentation time and mixing ratio of mungbean to soybean milk.

### Antioxidant capacity

**Figure 4:**
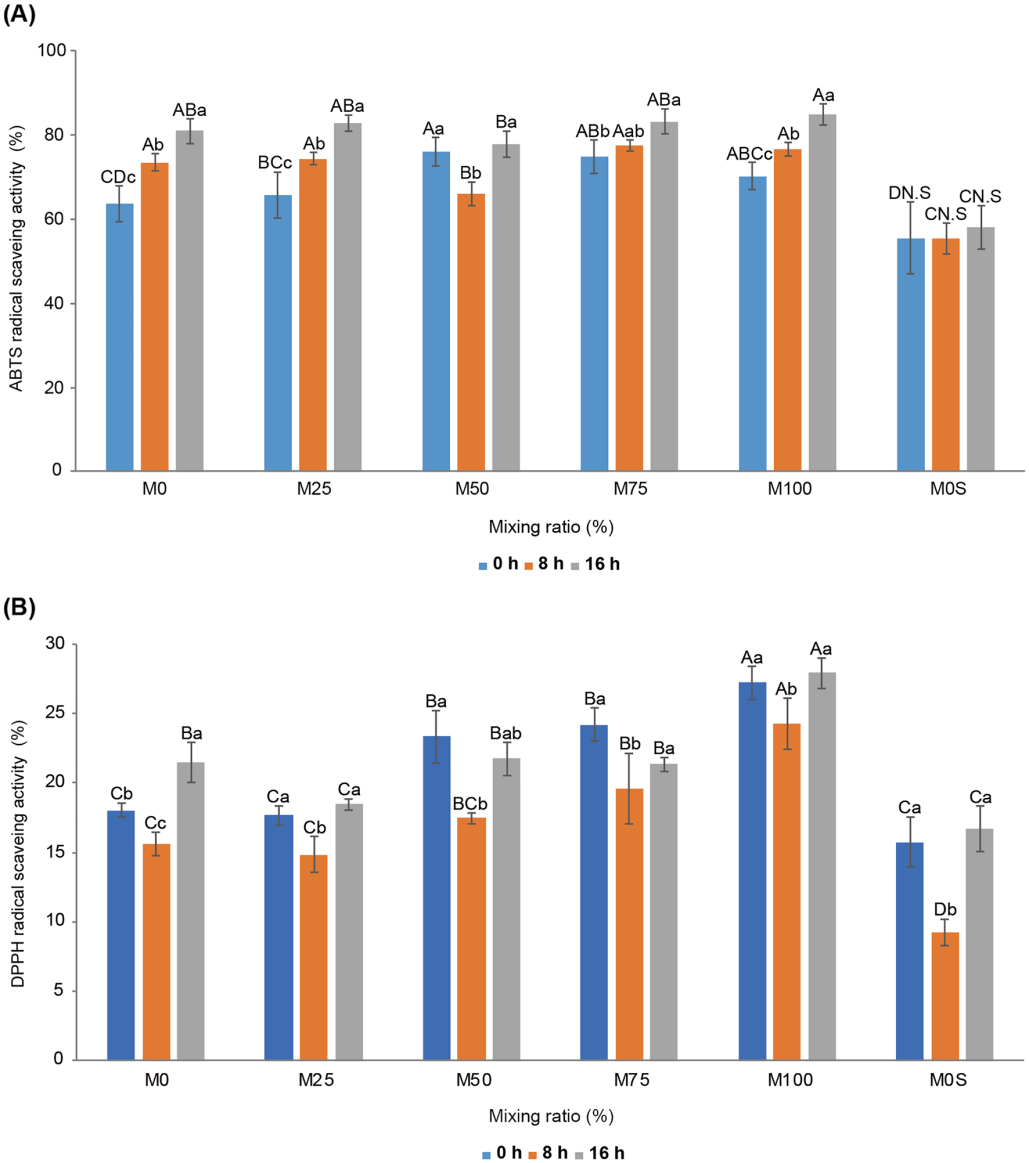
Changes in 2,2’-azino-bis-3-ethylbenzothiazoline-6-sulfonic acid (ABTS) and 2,2-diphenyl-1-picrylhydrazyl (DPPH) radical scavenging activity after fermentation of vegetable yoghurt according to the ratio of mungbean to soybean milk. M0, 25, 50, 75, and 100 represent the mungbean milk content (%), and M0S represents soybean milk 100% (mungbean milk 0%) with sucrose added. Fermentation times are represented by 0 h, 8 h, and 16 h. **A**–**D** represent significant differences in ABTS and DPPH radical scavenging activity of the samples according to each fermentation time, and a–c represent significant changes in ABTS and DPPH radical scavenging activity as the fermentation time of each sample increases.

The ABTS and DPPH radical scavenging activities according to the fermentation time of vegetable yoghurt with different mungbean and soybean milk contents were measured (Fig. 4A, B). In general, all samples showed an increase in ABTS radical scavenging activity during fermentation, and the sample added with mungbean milk showed higher DPPH radical scavenging activity than M0. M0S showed low antioxidant activity compared with the ABTS and DPPH radical scavenging activities of the samples without sucrose (M0, M25, M50, M75, and M100). M0S had no significant change in ABTS radical scavenging activity during fermentation. These results indicate that adding sugar can promote the activity of LAB, decrease pH, increase TA, and increase viscosity during fermentation but has no effect on the functional improvement of vegetable drinks.

### Sensory evaluation

**Figure 5:**
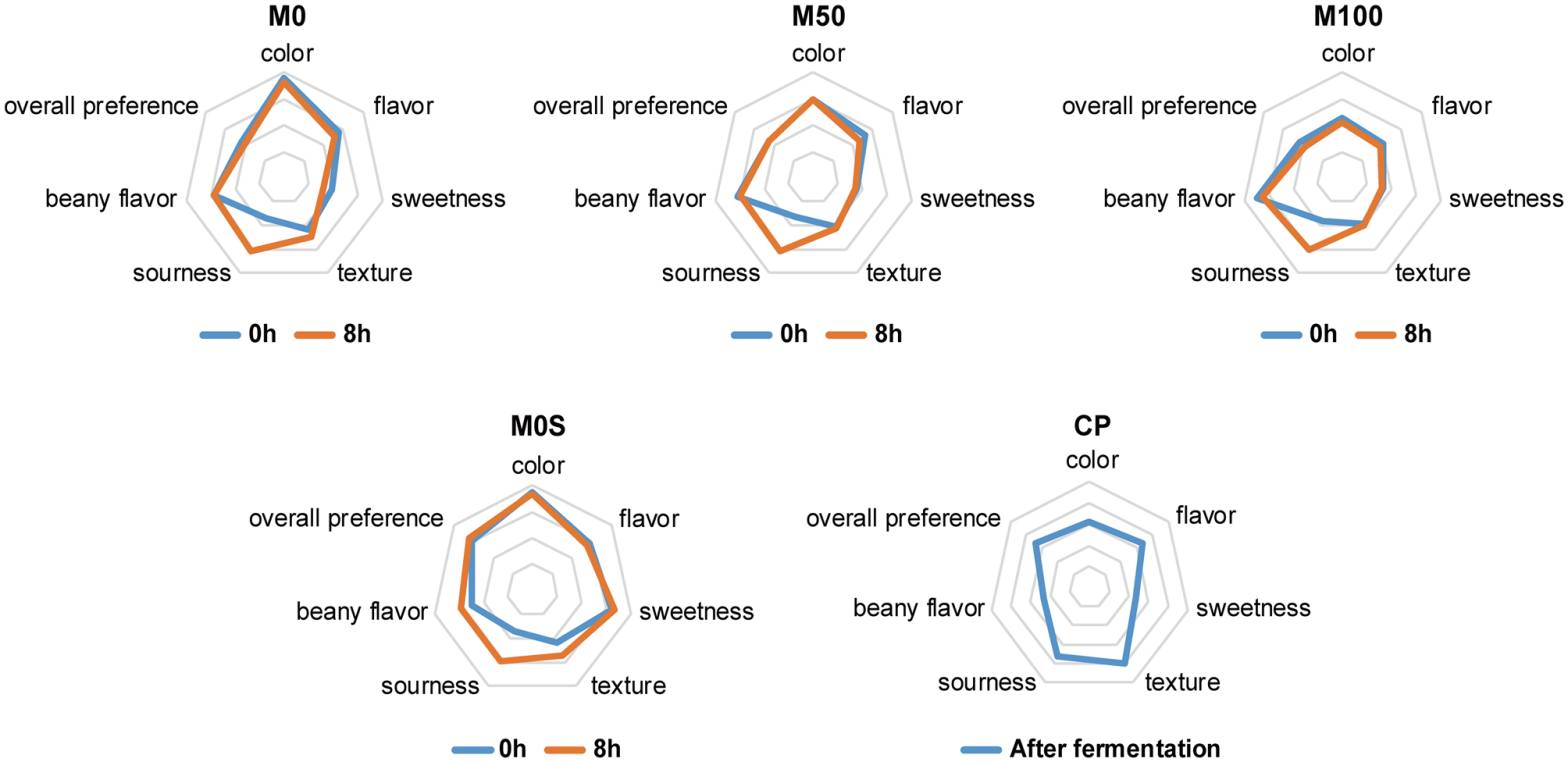
Sensory test of yoghurt with different mixing ratios of mungbean and soybean milk. M0, 25, 50, 75, and 100 represent the mungbean milk content (%), and M0S represents soybean milk 100% (mungbean milk 0%) with sucrose added. CP stands for commercial Greek soybean milk yoghurt. Fermentation times are represented by 0 h and 8 h. The test was conducted using a 5-point scale method with a minimum of 1 point and a maximum of 5 points for color, flavor, texture, sweetness, sourness, beany flavor, and overall preference.

Sensory tests were conducted for color, flavor, texture, sweetness, sourness, beany flavor, and overall preference of yoghurt with different ratios of mungbean to soybean milk, depending on the fermentation time (Fig. 5). The color preference decreased as the proportion of mungbean milk increased, with M100 showing the lowest score. The flavor of M100 fermented for 8 h was the lowest at 1.89 ± 0.98 points. The sweetness of M0 decreased significantly from 1.94 ± 1.01 points to 1.58 ± 0.84 points after fermentation, and the sweetness score was the highest for M0S. The textures of M50 and M100 were not significantly different before and after fermentation. The sourness of all samples (M0, M50, M100, and M0S) increased significantly after fermentation. The beany flavor increased as the proportion of mungbean milk increased. Overall, M0S after fermentation and commercial Greek soybean milk yoghurt (CP) scored the best. M100 had the lowest overall preference, probably because of its high acidity and beany flavor.

Fermentation by LAB can be used to improve organoleptic properties by reducing the unpleasant beany flavor^43,44^. However, in this study, the beany flavor was not improved by fermentation in most samples. Yi et al.^45^ reported that the sensory evaluation results varied when mungbean was fermented using *L. plantarum* 23169, *L. plantarum* 22699, and *L. plantarum* 2013 isolated from different sources. Fermentation using the *L. plantarum* 2013 strain reduced beany flavor and grass odor. In contrast, *L. plantarum* 22699 showed no differences in the beany flavor and grass odor compared to the non-fermented control group. The flavor resulting from fermentation varied unpredictably depending on the LAB strain. Therefore, to improve the overall preference for vegetable drinks with mungbean milk, adding natural sweeteners such as allulose should be considered.

This study evaluated the possibility of using mungbean milk as a LAB substrate. This study demonstrates that fermented mungbean milk is a potential food ingredient for developing milk substitutes that can improve the nutritional balance of dairy products and the possibility of mungbean milk as a substrate for LAB. The results of this study provide valuable information for producing vegetable drinks with high nutritional value.

### Supplementary Information

Supplementary Information.

## Data Availability

All data generated or analysed during this study are included in this published article and its supplementary information files.
