# Supplementary material for: The enhancement effect of mungbean on the physical, functional, and sensory characteristics of soy yoghurt
Source: Sci Rep. 2024 Feb 14;14:3684. doi: 10.1038/s41598-024-54106-9 (PMC10866981; doi:10.1038/s41598-024-54106-9)
Supplement: Supplementary file 1 — Supplementary Information. [file 41598_2024_54106_MOESM1_ESM.pdf]

The enhancement effect of mungbean on the physical, functional, and sensory characteristics of soy yoghurt

GYEONGSEON AN,[1] SUNGHOON PARK[2,3] & JUNGMIN HA[1,2*]

[1]Department of Plant Science, Gangneung-Wonju National University, Gangneung 25457, Republic of Korea

[2]Haeram Institute of Bakery Science, Gangneung-Wonju National University, Gangneung 25457, Republic of Korea

[3]Department of Food & Nutrition, Gangneung-Wonju National University, Gangneung, 25457, Republic of Korea

[*]Corresponding author : Jungmin Ha; (E-mail) j.ha@gwnu.ac.kr

Supplementary Table 1. Proximity analysis results before and after fermentation of mungbean and soybean milk.

Samples		Crude Protein (g/100 g)	Crude Fat (g/100 g)	Ash (g/100 g)
M0	0 h	2.90 ± 0.00[N.S]	1.65 ± 0.07[N.S]	0.40 ± 0.00[a]
	8 h	2.90 ± 0.00[N.S]	1.70 ± 0.00[N.S]	0.30 ± 0.00[b]
	16 h	2.90 ± 0.00[N.S]	1.70 ± 0.00[N.S]	0.30 ± 0.00[b]
M50	0 h	2.30 ± 0.00[N.S]	0.90 ± 0.00[N.S]	0.30 ± 0.00[N.S]
	8 h	2.30 ± 0.00[N.S]	0.90 ± 0.00[N.S]	0.30 ± 0.00[N.S]
	16 h	2.30 ± 0.00[N.S]	0.90 ± 0.00[N.S]	0.30 ± 0.00[N.S]
M100	0 h	1.70 ± 0.00[N.S]	0.10 ± 0.00[N.S]	0.20 ± 0.00[b]
	8 h	1.70 ± 0.00[N.S]	0.10 ± 0.00[N.S]	0.30 ± 0.00[a]
	16 h	1.70 ± 0.00[N.S]	0.10 ± 0.00[N.S]	0.20 ± 0.00[b]
M0S	0 h	2.80 ± 0.00[N.S]	1.30 ± 0.00[a]	0.40 ± 0.00[a]
	8 h	2.80 ± 0.00[N.S]	1.30 ± 0.00[a]	0.30 ± 0.00[b]
	16 h	2.80 ± 0.00[N.S]	0.90 ± 0.00[b]	0.30 ± 0.00[b]

*N.S: not significant.

M0, 25, 50, 75, and 100 represent the mungbean milk content (%), and M0S means soybean milk 100% (mungbean milk 0%) with sucrose added. Fermentation times are represented by 0 h, 8 h, 16 h. For nutritional analysis, crude protein, crude fat, and ash contents were measured for M0, M50, M100, and M0S after 0 h, 8 h, and 16 h of fermentation. Letters indicate statistically significant differences in crude protein, crude fat, and ash values using Duncan's multiple test (p < 0.05). a–c show significant changes in crude protein, crude fat, and ash values as the fermentation time of each sample increases.

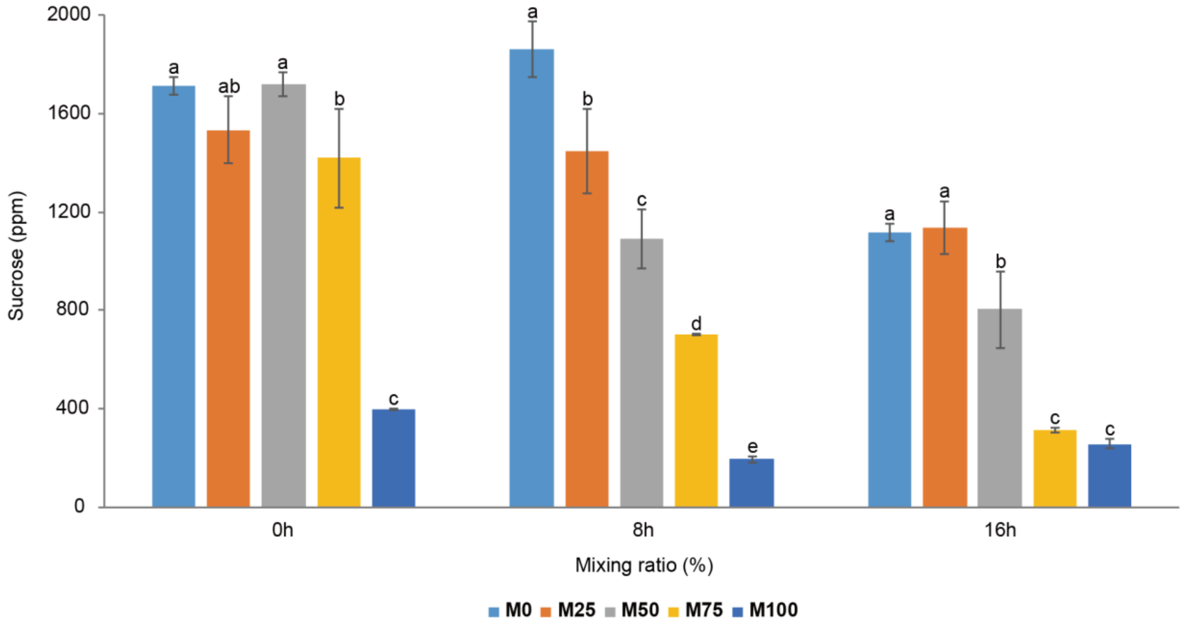

Supplementary Fig 1. Analysis of changes in sucrose in vegetable yoghurt before and after fermentation according to the ratio of mungbean to soybean milk. M0, 25, 50, 75, and 100 represent the mungbean milk content (%), and M0S means soybean milk 100% (mungbean milk 0%) with sucrose added. Fermentation times are represented by 0 h, 8 h, and 16 h. Letters indicate statistically significant differences in sucrose value using Duncan's multiple test (p < 0.05). a–c represent the significant sucrose difference of the samples for each fermentation time. The unit of sugar content is ppm.
